# Assessment of Socket Pressure during Walking in Rapid Fit Prosthetic Sockets

**DOI:** 10.3390/s22145224

**Published:** 2022-07-13

**Authors:** Kazuhiko Sasaki, Gary Guerra, Win Lei Phyu, Sirarat Chaisumritchoke, Prawina Sutdet, Sirintip Kaewtip

**Affiliations:** 1 Sirindhorn School of Prosthetics and Orthotics, Faculty of Medicine, Siriraj Hospital, Mahidol University, Bangkok 10700, Thailand; kazuhiko.sas@mahidol.edu (K.S.); win.phy@student.mahidol.edu (W.L.P.); sirarat.cha@mahidol.edu (S.C.); prawina.sut@mahidol.edu (P.S.); sirintip.kae@mahidol.edu (S.K.); 2 Department of Exercise and Sport Science, St. Mary’s University, One Camino Santa Maria, San Antonio, TX 78228, USA

**Keywords:** pressure sensor, lower limb prosthesis, amputee, outcome measure, polystyrene, prosthetics

## Abstract

(1) Background: A sustainable casting system that combines the use of a polystyrene bag, a prosthetic liner and a vacuum system was developed to reduce fabrication time while maintaining comfort for the trans-tibial prosthesis user. (2) Methods: Eight prosthetists (28.7 ± 8.25 years old) fit ten trans-tibial prosthesis wearers (46 ± 12.4 years old) with two types of total surface bearing (TSB) prostheses; a polystyrene bead (PS) prosthesis and a plaster of paris (POP) prosthesis. Duration of casting and combined mean peak pressure was measured at six locations on the residual limb using Force Sensing Resistors (FSR). A pressure uniformity score (%) was determined. Socket Comfort Scale (SCS) was also measured. (3) Results: Duration of casting for the POP method was 64.8 ± 9.53 min and 7.8 ± 2 min for the PS method, (*p* = 0.006). Pressure uniformity in the POP prosthesis was 79.3 ± 6.54 and 81.7 ± 5.83 in the PS prosthesis (*p* = 0.027). SCS in both prosthesis types were equivalent. (4) Conclusion: A rapid fit PS prosthesis was developed, with significantly shorter duration than the traditional POP method. Socket pressure uniformity was confirmed and improved in the PS method. Socket comfort was equal between the two prothesis types.

## 1. Introduction

Fabrication of lower limb prosthetic sockets is often a laborious time-consuming process. Traditional plaster of paris (POP), also called gypsum plaster, has been used for bandage casting and creating prosthetic positive plaster molds for decades; however, it necessitates extensive modification of the mold. POP is widely used in orthopedics and rehabilitation for low-cost one-off procedures. However, large amounts of material must be discarded as waste products which do not dissolve easily [1].

A recent trend in prosthetics has been to reduce these processes through the use of various rapid technologies. Digital scanners and three-dimensional printing are one such way; however, this is an expensive investment which precludes its use in resource-limited environments (RLE) [2]. In an attempt to address sustainability and remove the need for additional plaster model modification, we developed a sustainable casting system that combines a polystyrene bag, a prosthetic liner and a vacuum system. This polystyrene bead (PS) casting system negates the need for a POP bandage and modification of the plaster model. The liner and vacuum transmit necessary forces to the limb which automatically provide pre-modification for the final prosthetic socket.

Lower limb prosthetic sockets must provide adequate circumferential forces for the patient to bear weight, stand and walk comfortably [3]. The simplest manner of measuring prosthetic socket comfort is to ask the patient, using a socket comfort score scale (SCS) [4]. However, clinicians must be confident that once out of the clinic, the designed uniform socket forces will be distributed as evenly as possible to ensure prolonged comfort. Force Sensing Resistors (FSR) have been widely used to evaluate pressure occurring on amputees’ residual limbs [5] and aid exoskeleton design [6,7]. They have also been used to assist with gait-detection [8] and prosthetic hand development [9] and provide wearable designs [10]. The FSR is a thin film-like isometric force sensor whose electrical resistance reduces when stress is placed across the sensor area [11]. These sensors are small, low-cost, and robust [12,13], making them ideal for clinical evaluation of prosthetic sockets.

Our primary objective was to develop a PS casting system to reduce prosthetic fit time compared to the POP casting method. Moreover, to verify sufficient socket pressures in both PS and POP methods, a series of FSR sensors were used to measure residual limb socket pressures during walking.

## 2. Materials and Methods

This study was approved by the Siriraj Faculty of Medicine Institutional Review Board (Si 503/2018). All participants, both prosthetist as well as lower limb prosthesis users, provided informed consent prior to participating in the study. A total of 8 prosthetist (28.7 ± 8.25 years old) with average experience of 6.25 ± 8.31 years were recruited to cast 10 trans-tibial prosthesis wearers (46 ± 12.4 years old), height 167.1 ± 6.62 cm, and weight 74.5 ± 8.5 kg. Prosthesis users were classified as active prosthesis users (K3) [13] with either dysvascular, traumatic or congenital etiologies of amputation. Prosthesis users all had medium length round-shaped residua with no skin issues.

### 2.1. Participants

Each of the eight prosthetists had an opportunity to fabricate a prosthesis for at least one participant; however, two prosthetists created two prostheses. Prosthesis users were evaluated for a total surface bearing (TSB) trans-tibial prosthesis by using the POP and also the PS casting method. The TSB socket-style prosthesis was chosen because it permits uniformly distributed socket forces on the residual limb. This is a great improvement over the patella tendon-bearing (PTB) style socket which does not allow for hydrostatic control of the residual limb in the socket [14,15,16].

### 2.2. Prosthesis Fabrication

Prosthetists were randomly assigned to perform either POP or PS methods. For the POP method, the amputee was asked to sit in a chair while the prosthetist applied a 6 mm thermoplastic elastomer (TPE) gel liner (*ALPS* ELFR-XX-6, Easy Liner Super Stretch, St. Petersburg, FL, USA). Moist paster bandages were then wrapped circumferentially around the limb. Once the cast was set, it was removed and filled with a plaster water mix which quickly created a positive plaster model. This model was then modified by the prosthetist with the simple goal of reducing the overall circumference of the model by 4% per the guidelines [17]. Then, a rigid thermoplastic socket was fabricated, connected to a pylon tube with alignable components, an AERO prosthetic liner suspension [18], and a Solid Ankle Cushioned Heel (SACH) foot (Otto Bock Healthcare GmbH, Duderstadt, Germany). This was bench-aligned and fit with the patient for dynamic alignment until fitting optimization occurred.

**Figure 1:**
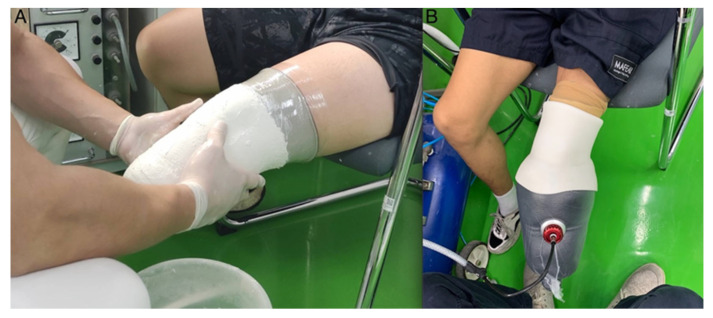
Image of (POP) plaster of paris and (PS) polystyrene bead casting methods. Note: (**A**) POP: plaster of paris casting; (**B**) PS: polystyrene bead casting.

Once the first prosthesis was completely fabricated, a second prosthesis using either the PS or POP method was created, depending on the prosthetist’s allocation. This rapid process required the amputee to sit while the 6 mm TPE gel liner was rolled onto the limb. Next, a polystyrene-bead-filled neoprene bag was rolled over the limb. The bead bag has a one-way valve and rubber hose attached, which were then connected to a vacuum system (Otto Bock Healthcare GmbH, D-37115, Otto Bock, Duderstadt, Germany). The prosthetist then turned on the vacuum system which was preset to provide a suction of (−70 kPa). Within seconds, the soft bead bag became rigid enough to acquire the residual limb’s surface features. This bag and liner were removed from the limb and filled with a plaster water mix to create a positive plaster model. There was no additional plaster modification needed, as the system combined the necessary forces on the residuum to account for a necessary 4% TSB socket reduction. Lastly, the prosthetist fabricated the rigid socket using Northplex^®^ (North Sea Plastics LTD, 099 Glasgow, UK) and pieced together a prosthesis using the exact same componentry and suspension used in the POP prosthesis. An image of both casting methods is shown in Figure 1. The time to complete the casting for the POP began at the donning of the limb sock and ceased once the rigid plaster cast was removed. The time to cast in the PS method began upon the donning of the limb sock and ceased once the PS cast was removed from the limb. Time to fabricate for both methods commenced the instant creation of the positive mold began for both the PS and the POP casts and ceased once the prosthesis was completely assembled. Fitting time began once the prosthetist initially placed the prosthesis on the participant and ceased once the participant exhibited an efficient gait. Recording the casting process started from when the liner was donned on the limb until removing the cast. Recording the modification process began at the point of removing the negative cast to acquiring the positive mold and lasted until the fitting was complete. Mean time was recorded for each participant to identify total method duration. Lastly, the participant’s residual limb was observed post-casting by both methods for any discoloration or issues.

### 2.3. Measurement Protocol

**Figure 2:**
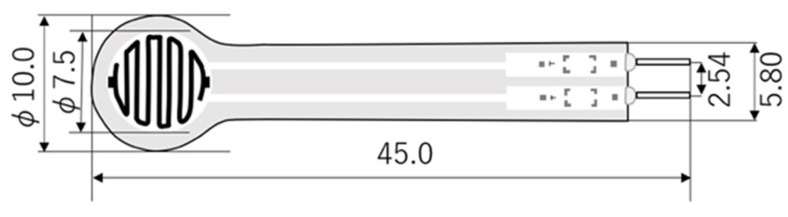
Schematic of Force Sensing Resistor sensor.

**Figure 3:**
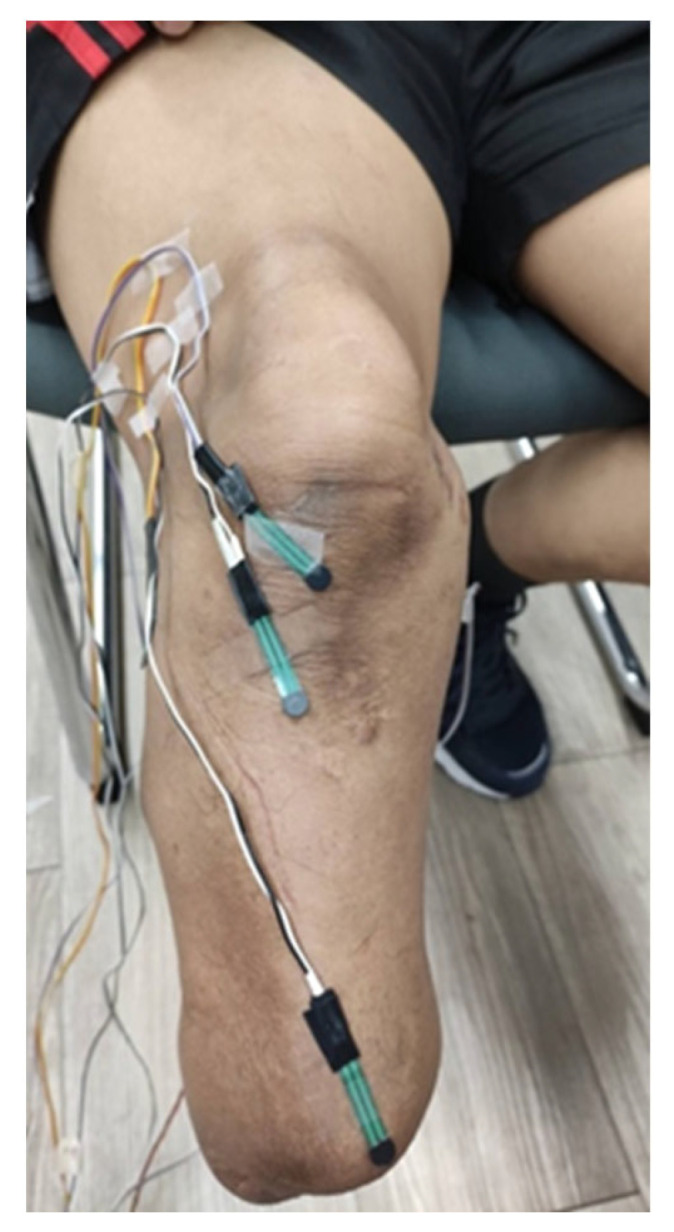
An example of force sensing resistor sensor placement on a residual limb.

Upon successfully fitting each of the two prostheses, each participant performed a 2 *min* treadmill walking trial. Participants were randomly assigned either a POP or a PS prosthesis for the pressure outcome measurement. Six 5 mm piezoresistive transducers FSR (Leanstar^®^, DF9-40, C406743, Suzhou, China) (Figure 2) were adhered to the residuum using medical grade tape to the patella tendon, the tibial tubercle, the anterior distal end of the tibia, the head of the fibula, the medial tibial flare and the posterior gastrocnemius (Figure 3). These locations were chosen as they are a mix of both pressure tolerant and pressure intolerant locations on an amputee’s limb [18]. However, sensors were first incrementally calibrated for accuracy with calibrated weights (100 g to 745 g). Results were compared to manufacturer results. An Arduino UNO board microcontroller with open-source Arduino *IDE* 2.0 *software* was used to record real-time sensor data in kilopascals (kPa) using a personal computer with Microsoft Windows 10 (Microsoft, Seattle, Washington, USA). Participants were asked to walk on a single-belt treadmill (Lode, Valiant, Lode B.V., The Netherlands) for two minutes at their self-selected walking speed (SSWS) (*0.65 m*/*s*) (*range 0.4–0.7* *m*/*s*)). A total of 40 steps sampled at 10 Hz were recorded [19]. Peak pressure within a step at each sensor for 40 steps was recorded. Immediately following the walking trial, participants were asked to score comfort of the socket using the SCS. This outcome measurement process was performed for each of the two prostheses amputees were provided and was administered by one study investigator. A video of the PS casting method is provided in the Supplementary Materials (Video S1: PS casting).

### 2.4. Statistical Analysis

Statistical analysis was performed using R statistical software version 4.2.0 (R Project for Statistical Computing) in RStudio statistical software version RStudio 2022.02.2 + 485 “Prairie Trillium”. A Wilcoxon Signed Rank Test was performed to explore for differences between the two casting methods on duration and pressure differences (*p* < 0.05). The combined mean peak pressure for all sensors and pooled standard deviation (SD) were calculated and used to calculate the coefficient of variation (CV). A lower CV indicated a lowered dispersion and greater uniformity (similarity) in the residual limb pressures. A higher CV indicated less uniformity in limb pressures. To simplify this concept, we applied an equation to the CV to determine ‘pressure uniformity’ (Equation (1)):

100 − (CV × 100)%,(1)

Thus, a score of 100% indicated complete pressure uniformity and 0% no uniformity. Socket comfort scores were analyzed using a two one-sided test (TOST) test of equivalency (*p* < 0.05). An equivalence margin of 10% was chosen [20]. This relates to an SCS difference of approximately 1.37 which is less than the minimum detectable change (MDC) of 2.7 [21].

## 3. Results

**Figure 4:**
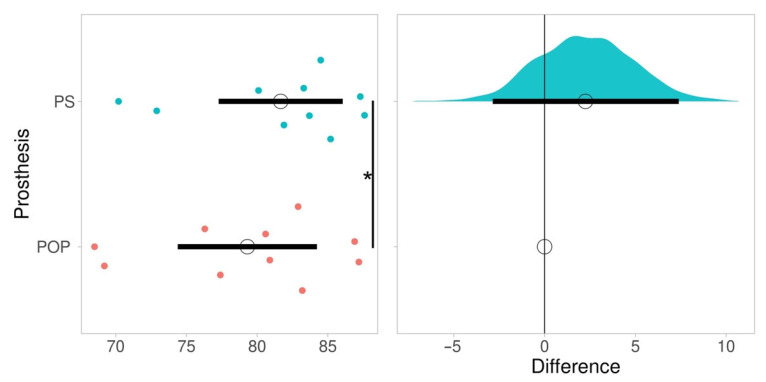
Plot of pressure uniformity between the two study prostheses. Note: PS: polystyrene bead prosthesis; POP: plaster of paris prosthesis; *: significant difference (*p* = 0.027). Mean of data is shown as an open circle Horizontal bar indicates 95% confidence interval. The plot on the right shows the effect size (difference), relative to the POP method. The bootstrap samples that are used to calculate the 95%CI of the effect size are shown as a distribution.

**Figure 5:**
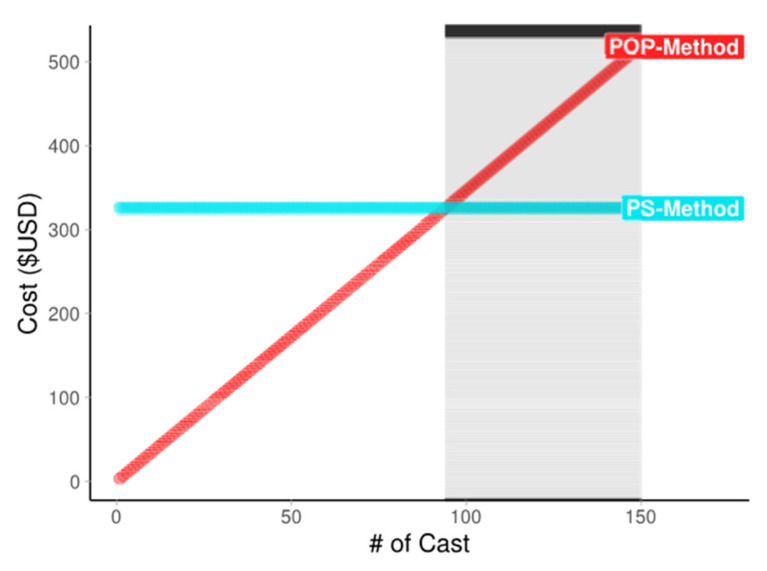
Plot illustrating initial and long-term costs of both casting systems. Note: #: number of cast, POP: plaster of paris, PS: polystyrene bead. # of cast: the number of casts and associated cost per cast from 0 to 150 prosthetic castings for both casting methods. Black horizontal line and gray shaded bar indicate the point at which additional POP castings become costlier than the PS casting method.

The duration of casting for the POP method was 64.8 ± 9.53 min and 7.8 ± 2 min for the PS method. This was a significant difference of approximately 50 min (*p* = 0.006). Fitting time in the POP method was 52.6 ± 8.64 min and in the PS method 48.6 ± 8.3 min. This difference was not significant (*p* = 0.414). No skin issues after casting were observed in any of the participants. Pressure uniformity in the POP prosthesis was 79.3 ± 6.54 and 81.7 ± 5.83 in the PS prosthesis (*p* = 0.027), Hedges’ *g* 0.038 (Figure 4). The equivalence test for SCS was significant, t(9) = −3.050, *p* = 0.008, Hedges’ *g* 0.09. A list of acronyms used in the study is provided in Abbreviations part. A figure illustrating the cost comparison of the two casting methods is provided in Figure 5.

## 4. Discussion

This study developed a rapid PS casting method for the trans-tibial prosthesis user. The duration of casting was significantly less than in the POP method, while fitting time was only slightly longer in the POP method. We employed FSR sensors to confirm uniformity of prosthetic socket fit for the two prosthetic fabrication methods. Residual limb pressure was successfully measured in both POP and PS prostheses, with participants using the PS prosthesis having significantly greater combined mean peak pressure than POP. SCS equivalency testing evidenced no differences between participant comfort in either PS or POP prosthesis. Moreover, SCS difference was less than the minimum detectable change for a true change in SCS and was 2.7 [21].

The prosthesis socket employed in this study was a TSB design that provided uniform circumferential pressure to the residuum. The TSB socket is the standard of care in developed settings. In addition, a suction suspension and an affordable roll-on AERO liner was employed. Taken together, these study prostheses exemplify the current standard of care in affordable prostheses. Moreover, this study is one of the first to objectively verify uniformity using sensors in a rapid fit TSB-style prosthesis. Using this PS bead bag and liner removed the need for plaster model modification in prosthetic fabrication. Previous scholars have employed the use of PS in casting however, these plaster casts still required plaster modification [22]. Furthermore, no objective outcome measurement of pressure on the residuum using the PS method had been previously performed. Outcome measurement is a critical feature of prosthetic treatments. The use of evidence-based practice necessitates the judicious use of outcome assessments to ensure comfort and function. Moreover, the advent of nano-bio sensors may offer advanced sensing solutions for clinicians [23]. As current FSR sensors can be cumbersome, fiber and textile-based sensors may soon offer unobtrusive wearable sensors for prosthetics [24,25,26,27]. Objective instruments such as the FSR offer a valid means to measure prosthetic socket interventions [28].

The cost of the PS casting system is approximately USD 350, and the one-time cost of the POP method is approximately USD 4.35. The POP casting method only necessitates 2–3 rolls of POP plaster wrap, whereas the higher price of the PS method is because of start-up purchases (pump USD 50–75, gel liner USD 200–250, hose USD 5, polystyrene bead bag USD 20). Although the PS system is expensive, this cost is a one-time materials purchase. In fact, the prosthetist can greatly reduce costs and improve sustainability by using an affordable ethyl–vinyl–acetate (EVA) liner instead of an expensive gel liner [29]. As sustainability in resource-limited environments is a key United Nations Sustainable Development Goal, it is imperative that both treatments and outcome measurement instruments be affordable [30]. The FSR sensors and Arduino technology make for an affordable clinical instrument.

This study is not without its limitations. Focusing on the cast duration outcome, additional prosthetists must be recruited to increase the study power. In addition, we recruited a variety of amputee participants for casting and thus did not control this variable. However, all amputees had medium length round-shaped residual limbs which were ideal for prosthesis fitting. In this study, sensors were placed over pressure-tolerant and intolerant areas. Pressure-tolerant areas can bear high pressure, whereas intolerant areas, such as the bony landmarks, are sensitive to weight bearing. Furthermore, this pressure measurement was restricted to the confines of a laboratory and did not consider free-living walking which may produce different socket pressures. However, the continued development of these sensors may offer a means for collecting whole limb, free-living prosthetic socket pressures in the future. In addition, we did not formally record the prosthetist feedback regarding the use of either system. As prosthetists are responsible for choosing the casting method, it is important to consider their preferences. Still, several of the prosthetists remarked on the ease and rapid procedures of the PS method.

This study is the first to use socket residuum pressures for the evaluation of the PS casting method creating prostheses. Both pressure measurement as well as PS casting method are straightforward and offer a streamlined alternative to trans-tibial prosthetic treatment.

## Data Availability

Not applicable.

## Supplementary Materials

The following are available online at https://www.mdpi.com/article/10.3390/s22145224/s1, Video S1: PS casting.

## Abbreviations

**Full Term**	**Abbreviation**
Total Surface Bearing	TSB
Polystyrene Bead	PS
Plaster of Paris	POP
Force Sensing Resistors	FSR
Socket Comfort Scale	SCS
Resource Limited Environments	RLE
Patella Tendon Bearing	PTB
Thermoplastic Elastomer	TPE
Solid Ankle Cushioned Heel	SACH
Kilopascal	kPa
Self-Selected Walking Speed	SSWS
Coefficient of Variation	CV
Minimum Detectable Change	MDC
Two One-Sided Test	TOST
Ethyl-Vinyl-Acetate	EVA
